# Acidophilic heterotrophs: basic aspects and technological applications

**DOI:** 10.3389/fmicb.2024.1374800

**Published:** 2024-05-17

**Authors:** Ernesto González, Fernando Vera, Felipe Scott, Cecilia Guerrero, Juan M. Bolívar, Germán Aroca, Jesús Ángel Muñoz, Miguel Ladero, Victoria E. Santos

**Affiliations:** ^1^Department of Chemical and Materials Engineering, Faculty of Chemistry, Universidad Complutense de Madrid, Madrid, Spain; ^2^School of Biochemical Engineering, Faculty of Engineering, Pontificia Universidad Católica de Valparaíso, Valparaíso, Chile; ^3^Faculty of Engineering and Applied Sciences, Universidad de Los Andes, Santiago, Chile

**Keywords:** acidophile, organic matter, heterotroph, mixotroph, acidiphilium, fermentation

## Abstract

Acidophiles comprise a group of microorganisms adapted to live in acidic environments. Despite acidophiles are usually associated with an autotrophic metabolism, more than 80 microorganisms capable of utilizing organic matter have been isolated from natural and man-made environments. The ability to reduce soluble and insoluble iron compounds has been described for many of these species and may be harnessed to develop new or improved mining processes when oxidative bioleaching is ineffective. Similarly, as these microorganisms grow in highly acidic media and the chances of contamination are reduced by the low pH, they may be employed to implement robust fermentation processes. By conducting an extensive literature review, this work presents an updated view of basic aspects and technological applications in biomining, bioremediation, fermentation processes aimed at biopolymers production, microbial electrochemical systems, and the potential use of extremozymes.

## 1 Introduction

Microorganisms are the earliest life forms that emerged on our planet nearly 3.7–4.3 billion years ago. Since then, living organisms have colonized the sea and land from pole to pole being nowadays omnipresent on Earth (Thakur et al., 2022). Although the concept of “extreme conditions” follows anthropocentric criteria rather than broader biological criteria, it is used to define the group of extremophiles (Merino et al., 2019). Hence, the term extremophile comprises a heterogeneous group of living organisms that thrive under extreme environmental conditions in harsh niches (Rampelotto, 2013). The heterogeneity found among extremophilic microorganisms, which might be the most abundant life forms on our planet, makes it necessary to establish subclassifications depending on the ability to grow at different pH, temperature, salinity, pressure, and water activity values (Thakur et al., 2022).

Regarding microorganisms able to grow at low pH, moderate acidophiles grow optimally from pH 3 to 5, whereas extreme acidophiles have an optimum pH at 3 or below. *Acidithiobacillus thiooxidans*, formerly known as *Thiobacillus thiooxidans*, was the first extreme acidophile discovered a century ago (Johnson and Quatrini, 2020). This bacterium is a mesophilic obligate aerobe that obtains energy from the oxidation of elemental sulfur and reduced inorganic sulfur compounds to sustain a strict autotrophic metabolism (Yang et al., 2019). Since then, several other extreme acidophilic autotrophs have been isolated from mine sites, hydrothermal vents, and geothermal acidic sites, including the most studied extremely acidophilic prokaryote *Acidithiobacillus ferrooxidans* (formerly named *Thiobacillus ferrooxidans*) (Quatrini and Johnson, 2019). Not surprising that acidophilic autotrophs have been utilized in coal and oil desulfurization, biotrickling, bioremediation, and biomining processes. Currently, they are a valuable tool for beneficiation of uranium, refractory gold, and low-grade copper ores due to their iron- and sulfur-oxidizing metabolism.

**Figure 1:**
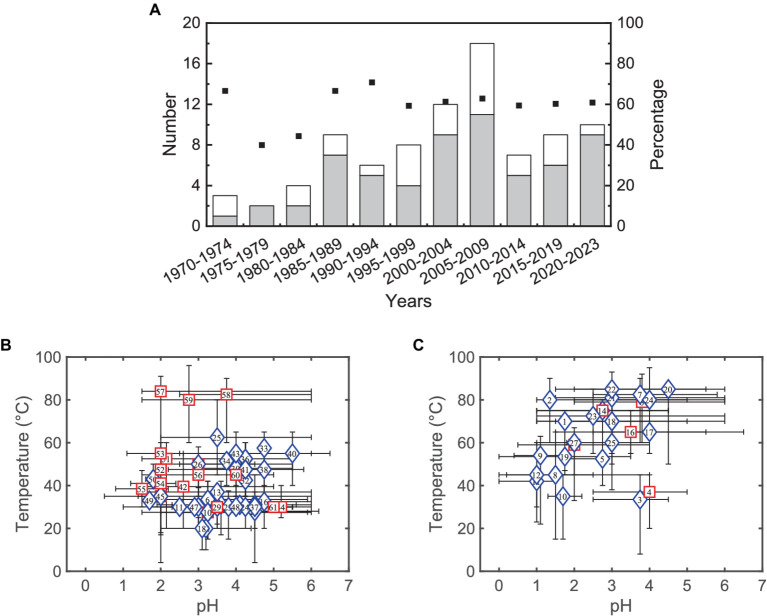
**(A)** Number of isolated acidophilic bacteria (gray bars) and archaea (white bars) able to degrade organic compounds, and the cumulative percentage of exclusive heterotrophic microorganisms (square markers). Panels **(B,C)** Growing conditions of acidophilic bacteria (Panel **B**) and archaea (Panel **C**) able to degrade organic compounds. The blue diamond represents an exclusive heterotrophic metabolism, and the red square shows a mixed heterotrophic/autotrophic metabolism. Markers have been plotted in the optimum values or in the center of optimal ranges. Supplementary Tables S1, S2 show detailed data while numbers in markers are referred to strains listed in both tables.

In contrast to acidophilic autotrophs, first extreme acidophilic heterotrophs were isolated in 1970 (Darland et al., 1970) and in the early 1980s (Harrison, 1981; Wichlacz and Unz, 1981; Johnson and Kelso, 1983), i.e., several decades after the discovery of *Acidithiobacillus ferrooxidans* and *Acidithiobacillus thiooxidans*. This may be paradoxical since heterotrophy is the most widespread form of metabolism among bacteria (Johnson and Roberto, 1997). Research in acidophiles is still highly focused on autotrophs, but several heterotrophic and mixotrophic acidophiles have been isolated over last decades (see Figure 1A). Figures 1B,C show that most bacteria grow better under mild and moderated temperatures, except those belonging to the genus *Sulfurisphaera*, while archaea tend to grow optimally at low pH or high temperatures. Since some microorganisms may be used in different bioprocesses, the aim of this article is to review and discuss significant and recent advances in the technological applications of organic matter-degrading acidophiles.

## 2 Basic aspects

Acidophiles use a variety of homeostatic mechanisms to maintain a circumneutral intracellular pH while living in acidic media (Baker-Austin and Dopson, 2007). Interestingly, acidophiles able to grow at extremely low pH (~pH 0, e.g., *Picrophilus oshimae*) can utilize organic matter as carbon and energy source (Xianke, 2021). As organic acids may act as uncouplers of the respiratory chain (Baker-Austin and Dopson, 2007), the ability to degrade them may be key to proliferate near pH 0 (Ciaramella et al., 2005). Most acidophilic heterotrophs degrade organic compounds using dissolved oxygen as final electron acceptor, while a strict respiratory metabolism has been reported for microorganisms belonging to the genera *Acidisphaera*, *Acidocella*, *Acidomonas*, *Alicyclobacillus*, and *Sulfobacillus* (Sievers and Swings, 2015; da Costa et al., 2015a,b; Hiraishi, 2015a,b). Notwithstanding, the utilization of alternative final electron acceptors such as Fe(III), Mn(IV), sulfate, and nitrate has been described for several strains: *Acidibacter ferrireducens, Acididesulfobacillus acetoxydans, Acidimicrobium ferrooxidans, Desulfosporosinus acidiphilus*, *Ferrimicrobium acidiphilum, Ferrithrix thermotolerans*, and some microorganisms belonging to the genera *Acidiphilium*, *Alicyclobacillus*, and *Sulfobacillus* (Johnson and McGinness, 1991; Wakao et al., 1994; Clark and Norris, 1996; Alazard et al., 2010; Falagán and Johnson, 2014; Norris, 2015a,b; da Costa et al., 2015b). In fact, dissimilatory iron reduction is a widespread characteristic among acidophilic heterotrophic bacteria (Coupland and Johnson, 2008), a metabolism that may derive from an ancient form of respiration when ferric iron was the most abundant oxidant (Knoll et al., 2016; Dong et al., 2021). However, a recent report showed that microbial iron reduction is usually reported at extreme pH and temperatures, but not when these extremes are combined; with the exception of four acidophilic hyperthermophiles (*Saccharolobus shibatae*, *Saccharolobus caldissimus*, *Saccharolobus solfataricus*, and *Acidianus manzaensis*) and two other strains (Nixon et al., 2022).

At circumneutral pH the solubility of Fe(III) is minimum (Cornell and Schwertmann, 2003), and some microorganisms (e.g., *Geobacter* and *Shewanella*) have developed several mechanisms for transferring electrons to ferric solids to act as electron sink (Gralnick and Newman, 2007). Nevertheless, iron reduction in acidophiles has not yet been sufficiently explored (Malik and Hedrich, 2022), considering the large availability of Fe(III) below pH 2.5, which solubility largely exceeds the solubility of oxygen in pure water at 25°C and 0.21 atm (2.56·10^−4^ mol/L) (Xing et al., 2014). Additionally, since acidophiles maintain a circumneutral intracellular pH, the energy harnessed from Fe(III) reduction (Equation 1) is similar to that obtained from using O_2_ as final electron acceptor (Equation 2). Redox transformations of other species can also be mediated by acidophilic heterotrophs, such as reduction of Cr(VI) (Cummings et al., 2007), reduction of Mo(VI) (Brierley and Brierley, 1982), or oxidation of As(III) (Battaglia-Brunet et al., 2011), but some of these reactions are not related to energy-conservation.

(1) $${Fe}^{3+}+e^{-}\to {Fe}^{2+}\;E^{{}^{\circ}\prime }\;\left(pH\;2\right)=0.77V$$

(2) $$O_{2}+4H^{+}+4e^{-}\to 2H_{2}O\;E^{{}^{\circ}\prime }\;\left(pH\;7\right)=0.81V$$

Some organic matter degrading acidophiles can synthesize pigments, such as bacteriochlorophyll and carotenoids. Bacteriochlorophylls are bacterial pigments involved in photosynthesis without the production of oxygen. To date, seven bacteriochlorophylls types have been identified with annotation using letters a–g (Yang et al., 2021). On the other hand, carotenoids (carotenes and xanthophylls) are tetraterpenes widely distributed in photosynthetic bacteria, and some species of archaea, fungi, algae, plants, and animals (Maoka, 2020). For instance, the genus *Acidiphilium* is characterized by the production of zinc-chelated bacteriochlorophyll a (Zn-BChl a) and the carotenoid spirilloxanthin (Hiraishi and Imhoff, 2015). However, *Acidisphaera rubrifaciens*, the only species belonging to the genus *Acidisphaera*, produces magnesium-chelated bacteriochlorophyll a (Mg-BChl a) as the main photopigment. Zn-BChl a is more stable than Mg-BChl a under acidic conditions (Hiraishi et al., 2000), and it has been shown to play a protectant role of the photosynthetic apparatus of *Acidiphilium rubrum* against copper toxicity (Jaime-Pérez et al., 2021). Biopolymers, such as polyhydroxyalkanoates (PHAs) and extracellular polymeric substances (EPS), are also produced by microorganisms belonging to the genus *Acidiphilium*. PHAs are biopolyesters accumulated by numerous microorganisms as storage compounds, being poly (3-hydroxybutyrate) (P3HB) the most common type of PHA (Palmeiro-Sánchez et al., 2022). Several strains of *Acidiphilium cryptum* can accumulate P3HB (Xu et al., 2013; González et al., 2023) while EPS production has also been reported for *Acidiphilium* sp. (Tapia et al., 2009, 2011; González et al., 2023). The section below addresses how these aspects may be involved in biotechnological applications of these microorganisms.

## 3 Technological applications

### 3.1 Biomining and bioremediation processes

Iron- and sulfur-oxidizing autotrophic acidophiles have been successfully used in mining applications. Therefore, several efforts have been made to use heterotrophic and mixotrophic acidophiles in biotechnology applied to this area. Most acidophiles able to reduce dissolved Fe(III) can also reductively dissolve ferric iron-containing minerals such as: amorphous ferric hydroxide, jarosite, magnetite, goethite, and hematite, among others (Johnson and McGinness, 1991; Bridge and Johnson, 1998, 2000; Hallberg et al., 2011; González et al., 2015a,b). Hence, the iron-reducing metabolism has been shown to be useful in biohydrometallurgical processes when oxidative bioleaching is ineffective or more sustainable methods are required (Malik and Hedrich, 2022). According to Eisele and Gabby (2014), iron-reducers may be used to: (i) remove iron impurities from materials where iron gives undesirable properties, (ii) recover iron from ores that are resistant to conventional processes, and (iii) promote the liberation and recovery of other metals.

The presence of iron impurities negatively affects the price of kaolin, bauxite, and silica due to color and other properties adversely affected. For example, main methods of kaolin bleaching comprises flocculation with polymers, chemical solubilization, extraction, and washing (Cobos-Murcia et al., 2022). Despite iron-reducing microorganisms can also be used for this purpose (Hosseini and Ahmadi, 2015; Yong et al., 2022), few studies report the utilization of heterotrophic acidophiles for kaolin bleaching (Kupka et al., 2007). On the other hand, metallic iron may be obtained from recalcitrant ores by the sequential using of iron-reducers (González et al., 2020) and electrowinning (Mostad et al., 2008). However, it seems not applicable at large scale because iron is highly abundant in the earth’s crust, and extractive metallurgy has developed highly optimized methods to obtain this metal at low cost.

Iron-reducing microorganisms can also be used for the recovery of metals different to iron. For example, manganese leaching from low-grade ores is increased by *Acidiphilium cryptum* growing heterotrophically (González et al., 2018), while rock phosphate, pyrite (Xiao et al., 2013), copper ores (Xu et al., 2010a; Yang et al., 2013), and printed circuit boards (Priya and Hait, 2020) are better leached when using mixed cultures of *Acidiphilium* sp. and *Acidithiobacillus ferrooxidans*. The action mechanism of iron reducers has been explained due to interactions with the solid compounds and other strains (i.e., iron- and sulfur-oxidizers) (Johnson and Roberto, 1997; Liu et al., 2011). Nevertheless, the ability of some heterotrophs to oxidize Fe(II) and sulfur-reduced compounds may also be relevant (Bacelar-Nicolau and Johnson, 1999; Priya and Hait, 2018; Bulaev, 2020; Panyushkina et al., 2021, 2022; Enuh and Aytar Çelik, 2022; Cho et al., 2023).

In this regard, a mixed culture of *Acidiphilium multivorum* and *Acidithiobacillus ferrooxidans* has been used in a biomineralization process where iron and sulfate are removed from acid mine drainage as schwertmannite to decrease the subsequent lime consumption and sludge generation (Jin et al., 2020). Similarly, a mixed culture of *Alicyclobacillus tolerans* and *Acidiphilium cryptum* was able to produce crystalline schwertmannite precipitates with the potential to remove arsenic from acidic effluents (Nural Yaman et al., 2023). Other applications related to highly impactful topics in material sciences include leaching of rare earths elements (e.g., by *Acidomonas methanolica*) (Hedrich et al., 2023), and the production of precious metals nanoparticles (palladium, gold and platinum, e.g., by *Acidocella aromatica* and *Acidiphilium cryptum*) (Okibe et al., 2017; Rizki and Okibe, 2018; Matsumoto et al., 2021).

### 3.2 Fermentation processes

Microbial fermentation is currently used for producing food and beverages, food ingredients and supplements, pharmaceuticals and nutraceuticals, monomers, solvents, and biofuels. The utilization of extremophiles in fermentation processes has been encouraged in recent works to increase the competitiveness of these processes (Chen and Jiang, 2018; Chen and Liu, 2021), having PHA as a particular product which can be generated using extremophiles (Koller, 2017). The accumulation of P3HB by acidophilic heterotrophs was presumed or reported in some early works where TEM imaging showed electron-transparent granules (Wichlacz et al., 1986; Matsuzawa et al., 2000). However, early studies in which the intracellular polymer was extracted and analyzed were first published by Yang et al. (2007) and Peng et al. (2008). *Acidiphilium* sp. DX1-1 (Zhang et al., 2013) has been the most commonly used strain to study the accumulation of P3HB at low pH from glucose, although the type strain *A. cryptum* Lhet 2 was utilized in a recent study using glycerol (González et al., 2023). The optimal conditions for P3HB production (glucose 40 g/L, KNO_3_ 15 g/L, and pH 3.0) were determined through an orthogonal array test which yielded the maximum of 19.75 g/L of P3HB (Xu et al., 2008). On the other hand, Xu et al. (2010) reported that chloroform-sodium hypochlorite was the best method for extraction of P3HB from *A. cryptum* (73% extraction and 92% purity) to obtain a material with a crystallinity degree of 46% formed mostly by fragments of 672 Da.

The expression of 13 genes related to the metabolism of P3HB was studied under different C:N ratios using real-time PCR (Xu et al., 2010b). This work showed upregulation of these genes when *A. cryptum* was grown using a C:N ratio equal to 2.4 to obtain 0.88 g of P3HB per gram of dry cells. Hence, acetyl-CoA synthetase and poly-β-hydroxybutyrate polymerase were pointed as the most upregulated genes for P3HB synthesis under the optimal C:N ratio. The authors also suggest that the P3HB yield may be raised using molecular biology techniques to increase the expression of *Acry_3030* (poly-β-hydroxybutyrate polymerase) or decrease the expression of *Acry_2759* (polyhydroxyalkanoate depolymerase) since there are no side pathways to polymerize or depolymerize P3HB, a task that may be addressed by synthetic biology of extremophiles (Ye et al., 2023).

The accumulation of P3HB and the expression of genes related to P3HB synthesis, CO_2_ fixation, and sulfur metabolism were studied in media containing glucose, elemental sulfur and mixtures of both substrates (Xu et al., 2013). The values of P3HB accumulated at the stationary phase are atypical when compared to other studies because, in most cases, they are higher than the values attainable by the initial concentration of glucose, being these results attributed to fixation of atmospheric CO_2_ through the Calvin cycle (Li et al., 2020). The highest overexpression of genes related to CO_2_ fixation was detected in the culture performed in medium containing glucose 1 g/L and elemental sulfur 5 g/L, a condition that produced 6.2 g/L of P3HB at the stationary phase. On the other hand, the highest overexpression of genes related to P3HB accumulation was observed in media containing 5 g/L of sulfur and 5 or 10 g/L of glucose, conditions that yielded the highest P3HB accumulations reported in this work, 8.3 or 14.1 g/L of P3HB at the stationary phase, respectively. Although these results seem promising for obtaining an efficient process for transforming CO_2_ into bioplastics (Kajla et al., 2022), to the best of our knowledge there are no subsequent studies using media containing sulfur and other organic compounds/residues, or addressing the up-scaling of this process.

Polyhydroxyalkanoates are not the only polymers synthesized by extremophiles. Nicolaus et al. (1993) showed that *Sulfolobus solfataricus* MT3 and MT4 synthesize a soluble exopolysaccharide when grown at 75 and 88°C, respectively. The analysis performed on these exopolymers showed the presence of glucose, mannose, glucosamine, and galactose in proportion 1.2:1.0:0.77:0.73 and 1.2:1.0:0.18:0.13 for MT3 and MT4 strains, respectively. More recent studies used lectin staining to show the presence of galactose, glucosamine and mannose/glucose residues in the extracellular polysaccharide synthesized by attached cells of *S. solfataricus, Sulfolobus tokodaii* and *Sulfolobus acidocalcarius* (Koerdt et al., 2010; Zolghadr et al., 2010). *Acidiphilium* sp. also produces bound EPS mainly consisting of proteins and carbohydrates where Fe(III) can be sorbed (Tapia et al., 2009, 2011), whereas *A. cryptum* Lhet 2 generates soluble EPS which analysis showed the presence of mannose, rhamnose, and glucose in a proportion near to 3.2:2.3:1 (González et al., 2023).

### 3.3 Microbial electrochemical systems

Microbial electrochemical systems are devices where microorganism mediate electrochemical reactions by exchanging electrons with an electrode through direct or indirect mechanisms (Chaudhary et al., 2022). The acidophilic bacterium *Acidiphilium cryptum* Lhet2 has been used for electricity generation in a microbial fuel cell operating at low pH (≤4.0) (Borole et al., 2008). The presence of dissolved iron in the medium enables the current generation. However, supplementation with a chelating agent (nitrilotriacetic acid) and an electron shuttle (phenosafranin) led to a higher steady-state voltage output. Although the maximum power density obtained (12.6 mW/m^2^) was low when compared to the maximum known values (5.61–7.72 W/m^2^) (Slate et al., 2019; Ren, 2021), the utilization of a acidophilic strain in optimized systems (e.g., miniaturized devices) may prevent the anode acidification; a phenomenon that inhibits the microbial activity by accumulation of hydrogen ions (Obileke et al., 2021).

*Acidiphilium* sp. strain 3.2 Sup 5 has also been reported to be an electrogenic strain able to produce currents between 2.0 and 3.0 A/m^2^ when degrading glucose at pH 2.5 (Malki et al., 2008). In this study, the current was reduced by ~25% when the colonized electrode was moved to a new glucose solution free of cells, thereby indicating that the attached cells are mainly responsible for the stablished current. Nonetheless, this bioanode seems to be highly resilient to oxygen infiltration in the anodic chamber because polarization curves obtained in the absence and presence of dissolved oxygen (6.2 ppm) were similar. The draft genome of a third electroactive bacteria belonging to the *Acidiphilium* genus has also been reported (San Martin-Uriz et al., 2011). Malki et al. (2008) suggested that the mechanism for electron transfer is via redox proteins allocated on the bacterial membrane or via excreted redox compounds. However, a more recent study reported that *Acidiphilium cryptum* JF-5 can form extracellular appendages, although the electrical conductivity of these appendages was not tested (González et al., 2018).

Previous studies have examined mesophilic electrogenic microorganisms, but only recently a thermophilic electrogenic bacteria phylogenetically related to *Alicyclobacillus hesperidum* was isolated from a microbial fuel cell (Zhang et al., 2021). This bacterium was able to grow at pH 3.0 and 50°C generating a maximum power density of 188.1 mW/m^2^. The authors proposed the self-excretion of soluble redox-active small molecules, such as quinones, as the mechanism for electron transfer for this bacterium. Few studies have addressed the electrochemical properties of acidophilic heterotrophs, but we speculate that there are probably several other electroactive microorganisms able to proliferate under similar conditions. Our assumption is based on the facts that (i) almost all organisms that can catabolically reduce ferric iron are also able to reduce an anode surface (Richter et al., 2012), and (ii) dissimilatory iron reduction is a widespread ability among acidophilic heterotrophic microorganisms (Coupland and Johnson, 2008).

### 3.4 Extremozymes

Extremozymes are enzymes obtained from extremophiles that can be used to catalyze reactions under extreme conditions, owing the prevalence of acidic amino acids on the protein surface (Samanta et al., 2022). Since intracellular enzymes of acidophiles operate at circumneutral pH (Baker-Austin and Dopson, 2007), it may be expected that only extracellular enzymes are resistant to low pH. However, several studies have reported that certain intracellular enzymes are capable of operating at extremely low pH (Golyshina et al., 2006). Extremozymes of interest that are tolerant to low pH include amylases, glucoamylases, xylanases, cellulases, proteases, and oxidases. These extremozymes may be useful in biofuel production, food mining, starch processing, desulfurization of coal, valuable metal recovery, and feed component (Samanta et al., 2022).

Several polysaccharide-degrading enzymes (e.g., α- and β-glucosidase, endoglucanase, and mannanase) have been isolated from acidophiles. For example, She et al. (2001) sequenced the genome of *Sulfolobus solfataricus* and found three genes encoding potentially secreted endo-β-glucanases (*sso1354*, *sso1949*, and *sso2534*). Later studies showed that the endo-β-glucanase Sso2534 is active at pH 5.8, the protein Sso1354 works optimally at pH lower than 4.5, and the protein Sso1949 has optimum conditions at pH 1.8 and 80°C (Limauro et al., 2001; Huang et al., 2005; Girfoglio et al., 2012; Lalithambika et al., 2012). In addition, the endoglucanase CelA4 produced by *Alicyclobacillus* sp. works optimally at 65°C and pH 2.6, being stable over a wide pH range (1.8–7.6) and resistant to acidic and neutral proteases (Bai et al., 2010). Boyce and Walsh (2018) showed that *Sulfolobus shibatae* produces an endo-1,4-β-glucanase which has its maximum activity at 95–100°C and pH in the range 3.0–5.0. This enzyme was able to hydrolyze barley β-glucan, lichenin, CMC, and xylan.

Three intracellular α-glucosidases of *Ferroplasma acidiphilum* exhibit no similarity to other glycosyl hydrolases (Golyshina et al., 2006). The optimal temperature for these enzymes is 60°C and their optimal pH is in the range 2.0–4.0, values significantly lower than the intracellular pH (5.6). On the other hand, *Sulfolobus acidocaldarius* produces a β-glucosidase belonging to the GH1 family (Park et al., 2010). This enzyme operates optimally at pH 5.5 and 90°C, although its half-life increases from 0.2 to 494 h when the temperature decreases from 90 to 70°C. Mannanases of microbial origin are mainly secreted extracellularly, although intracellular mannanases are produced by few bacteria (e.g., *Alicyclobacillus acidocaldarius*). The endo-β-1,4-mannanase produced by this acidophile has significant transglycosylation activity and relatively low hydrolytic activity, working optimally at pH 5.5 and 65°C (Zhang et al., 2008).

A recent study isolated carboxylesterases from the microbial community inhabiting an acid mine drainage (pH ~2) (Vidal et al., 2022). In this work, 16 esterases were identified in microorganisms belonging to the genera *Acidithrix*, *Acidimicrobium*/*Ferrimicrobium* and *Acidiphilium*, among others, being 10 of them successfully expressed in *E. coli*. The results showed that optimal pH and temperature were in the ranges 7.0–9.0 and 30–65°C, respectively, although at pH 5.5 the enzymes retained 33–68% of their activity. Six of these hydrolases showed efficient degradation of acrylic- and terephthalic-like esters, which may be relevant for degradation of plastics. Esterases of *Ferroplasma acidiphilum* (Golyshina et al., 2006) and *Acidiphilium* sp. (Isobe and Wakao, 1996) have also been reported. The former exhibits excellent activity near pH 2 despite being intracellularly located, whereas the latter, located both in cells and culture supernatant, is active at pH 4.0–5.0 hydrolyzing Tween 80. Other enzymes can also be obtained from this microbial group, having as example the histidine ammonia lyase from *Thermoplasma acidophilum* which was used to implement a microreactor able to operate at pH 2.8 (Ade et al., 2022). Additionally, Ortiz-Cortés et al. (2021) showed the presence of β-galactosidase, cellulase, lipase, xylanase, and protease activities in the cell-free medium obtained after culturing *Alicyclobacillus* sp. at pH 3 and 5, while Micciche et al. (2020) identified mercury and arsenic reductases in some *Acidiphilium* strains.

## 4 Concluding remarks and future perspectives

While autotrophic acidophiles, like *Acidithiobacillus ferrooxidans*, have played pivotal roles in the understating of life at low pH and some bioprocesses, heterotrophic and mixotrophic acidophiles have been significantly less studied. Notwithstanding, by conducting an extensive literature review, this work presented an integrative view of the basic metabolic and culture aspects of heterotrophic acidophiles. These traits lay at the core of the development of technological applications, including biomining, bioremediation, and fermentation processes aimed at biopolymers production, microbial electrochemical systems, and the potential use of extremozymes.

Although acidophiles capable of utilizing organic matter may be relevant to establish new or optimized mining processes, special attention should be paid into (i) the proliferation of undesired microorganisms in liquid media and (ii) the availability and price of required organic matter. Despite sterilization of base metal ores is probably unfeasible from an economic point of view, some strains (e.g., *Acidithiobacillus ferrooxidans*) or microbial groups (e.g., iron and sulfur oxidizing chemolithoautotrophs) may be inhibited by organic compounds (Määttä et al., 2022), or by controlling the osmotic pressure and concentration of certain ions (Harahuc et al., 2000). On the other hand, problems related to the availability and price of organic matter may be confronted using cheap and locally available organic substrates, having as example the domestic effluent used by Magowo et al. (2020) to drive sulfate reduction in an acid mine drainage.

Acidophilic heterotrophs also constitute a good option to establish robust fermentation processes as the culture proceeds self-protected by the low pH (Chen and Jiang, 2018). Regarding production of PHAs, significant accumulations have been reported at high salinity and extreme temperatures (Koller, 2017), but acidophiles have been not extensively studied for this purpose. Hence, more studies aimed to modify their metabolism and optimize culture parameters will be required to achieve large efficiencies. Finally, the utilization of acidophilic heterotrophs in microbial electrochemical systems and identification of useful extremozymes on them are fields even newer or less explored than those previously reported. Hence, probably bioinformatics and synthetic biology will be valuable tools to harness their potential.

## Author contributions

EG: Writing – review & editing, Writing – original draft. FV: Writing – original draft. FS: Writing – original draft. CG: Writing – original draft. JB: Writing – review & editing. GA: Writing – review & editing. JM: Writing – review & editing. ML: Writing – review & editing. VS: Writing – review & editing.
