# Supplementary material for: Acidophilic heterotrophs: basic aspects and technological applications
Source: Front Microbiol. 2024 May 17;15:1374800. doi: 10.3389/fmicb.2024.1374800 (PMC11141062; doi:10.3389/fmicb.2024.1374800)
Supplement: Supplementary file 1 [file Table_1.DOCX]

Reviewer 2:

This article presents a comprehensive review of acidophiles and their technological applications. Even though the review fills an important gap and is well-structured, there is always room to improve the manuscript. Here are some recommendations:

R: We appreciate the comments made the reviewer 2.

The title does not fit well. Please improve it. The section “capable of utilizing organic matter” is part of the study but not the main focus.

R: The title was changed to “Acidophilic heterotrophs: basic aspects and technological applications”.

Please add a sentence summarizing the bibliometric study you did in the manuscript. Which sources did you get all these references from? Which keywords did you use for this search? How many experimental articles and reviews are about this topic? This will help to stress the importance of this review. Avoid old references.

R: The bibliometric study was performed using the following keyword: (acidophilic or acidophile) and (heterotroph or heterotrophic or organic matter or organic compound). Obtained results were manually checked to (1) determine which genera were described as acidophilic heterotrophs or mixotrophs and (2) discard non relevant works. The list of genera was complemented using published lists of microorganisms environmentally relevant in acidic environments. Strains belonging to listed genera were checked at the List of Prokaryotic names with Standing in Nomenclature (<https://lpsn.dsmz.de/>) and the Bergey's Manual of Systematics of Archaea and Bacteria. Information relative to isolation, culture conditions (pH and temperature) and applications was obtained from the Bergey's Manual of Systematics of Archaea and Bacteria and scientific publications. Some old references were included when they report the first isolation or include relevant information. This information was not included in the manuscript as the current version slightly exceeds the word limit for a Mini Review article.

Please add a figure that summarizes the technological applications.

R: A figure summarizing the technological applications was not included in this new version as the authors think it does not contribute substantially to the work.

Please summarize the table you want to add to the main manuscript and move the big table to supplementary materials.

R: The table was moved to supplementary materials. The new version includes a figure summarizing some of the information.

Reviewer 3:

The manuscript provides an overview of the technological applications of acidophilic microorganisms, particularly focusing on their roles in biomining and bioremediation processes, fermentation, microbial electrochemical systems, and the production of extremozymes. The review presents information on the metabolic capabilities, enzymatic activities, and potential industrial applications of acidophilic microorganisms.

R: We appreciate the comments made the reviewer 3.

While the manuscript briefly mentions future perspectives in the concluding remarks, it lacks an in-depth discussion on the challenges associated with harnessing acidophilic microorganisms for biotechnological applications.

R: The section “Concluding remarks and future perspectives” was improved to include a discussion of the challenges.

The manuscript contains several grammatical errors, awkward phrasings, and unclear passages, which can hinder the readability and understanding of the content. Improving the clarity and coherence of the writing would enhance the overall quality of the manuscript. I would suggest that the authors seek the services of a language editor to aid them.

R: The manuscript was entirely revised. Awkward and unclear phrases were changed, and grammatical errors were corrected.
